# A prospective seroepidemiological study of toxocariasis during early childhood in coastal Ecuador: potential for congenital transmission and risk factors for infection

**DOI:** 10.1186/s13071-020-04575-4

**Published:** 2021-02-05

**Authors:** Aida Y. Oviedo-Vera, Irina Chis Ster, Martha E. Chico, Marcia B. Silva, Luis F. Salazar-Garcés, Neuza M. Alcantara-Neves, Philip J. Cooper

**Affiliations:** 1 Fundacion Ecuatoriana Para Investigación en Salud, Quinindé, Ecuador; 2 Institute of Health Sciences, Federal University of Bahia, Salvador, Bahia Brazil; 3 Institute of Infection and Immunity, St George’s University of London, Cranmer Terrace, London, SW17 0RE UK; 4 Escuela de Medicina, Universidad Internacional del Ecuador, Quito, Ecuador

**Keywords:** *Toxocara* spp., Birth cohort, Congenital transmission, Seroprevalence, Seroconversion, Risk factors, Childhood, Ecuador

## Abstract

**Background:**

Although *Toxocara* spp. infection has a worldwide distribution, to our knowledge, no data from birth cohorts have been reported in published studies on the potential for congenital transmission and determinants of infection in early childhood.

**Methods:**

We followed 290 mother-infant pairs from birth to 5 years of age through periodic collection of data and samples at birth, 7 and 13 months and 2, 3 and 5 years of age. Data on potential risk factors and confounders were collected by maternal questionnaire. Blood for plasma was collected from the mother at time of birth and periodically from the child for detection of anti-*Toxocara* spp. immunoglobulin G (IgG) antibodies using a *Toxocara canis* larval excretory-secretory antigen-based enzyme-linked immunosorbent assay. Stool samples were collected from the mother around the time of birth and periodically from the child for microscopic detection of soil-transmitted helminths (STH). Associations between potential risk factors and *Toxocara* spp. seroprevalence and seroconversion were estimated using multivariable logistic regression and generalized estimating equations.

**Results:**

*Toxocara* spp. seroprevalence was 80.7% in mothers and in children was 0%, 9.3%, 48.4%, 64.9%, and 80.9% at 7 months, 13 months, 2, 3 and 5 years, respectively. Risk factors significantly associated with increases in seroprevalence over the first 5 years of life in multivariable analyses were age [Odds ratio (OR) 2.06, 95% confidence interval (CI) 1.39–2.27, *P* < 0001], male sex (female vs. male: OR 0.66, 95% CI 0.48–0.89, *P* = 0.006), maternal ethnicity (non-Afro vs. Afro-Ecuadorian: OR 0.65, 95% CI 0.47–0.91, *P* = 0.011), lower maternal educational and socioeconomic level, and childhood STH (OR 2.29, 95% CI 1.51–3.47, *P* < 0.001). Seroconversion rates for infection were greatest at 2 years of age (3.8%/month). Factors associated significantly with seroconversion at 2, 3 or 5 years were childhood STH infection, male sex, and more frequent domestic cat exposure.

**Conclusions:**

Our data, from an area of high *Toxocara* spp. endemicity, indicate no congenital transmission but high rates of seroconversion after 13 months of age reaching maternal levels of seroprevalence by 5 years of age. Factors associated with seroprevalence and seroconversion included STH infections, domestic cats, maternal ethnicity, male sex, STH infections, and markers of greater poverty.
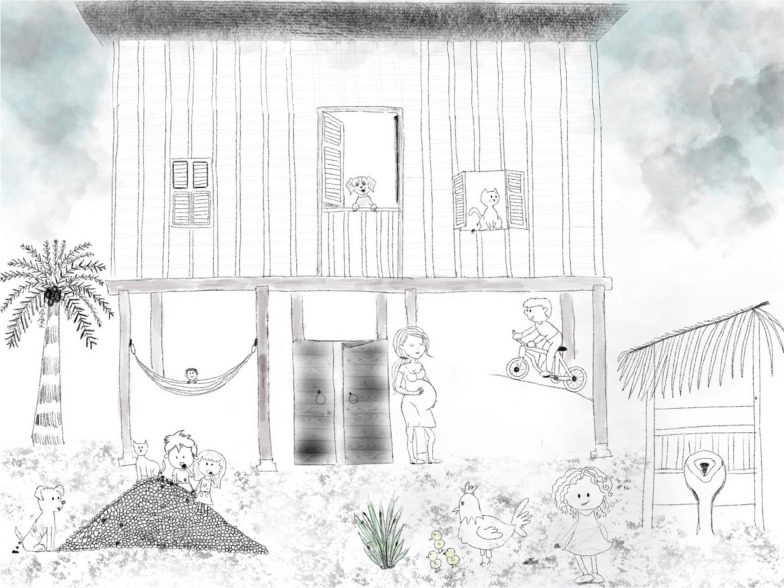

## Background

Toxocariasis is a common parasitic disease with a worldwide distribution [1]. *Toxocara* spp. infection affects particularly children in tropical regions living in conditions of poverty and poor hygiene among whom seroprevalence rates often exceed 50% [1]. *Toxocara* spp. include *Toxocara canis* and *Toxocara cati* that parasitize the small intestine of dogs and cats, respectively. Humans and several animals are paratenic hosts, infected through accidental ingestion of embryonated eggs through contaminated water, soil, and food. *Toxocara* spp. cannot develop in humans beyond the larval stage, although larvae may survive migrating in tissues for months or years [1].

While most human infections are asymptomatic, infection may have severe clinical consequences causing visceral larval migrans or infections of the central nervous system including the eyes [1]. Disease severity is considered to depend on larval burden and host inflammatory response to invasive larvae [1].

It has been suggested that congenital infections may arise from larval invasion of the placenta from a parasitized mother [2, 3]. However, there is no definitive evidence of congenital transmission in humans, although congenital infections have been demonstrated in other paratenic hosts such as mice [4]. There are few data documenting the epidemiology of toxocariasis from birth in human populations [1]. We used data and samples from a birth cohort in Ecuador to study the potential for congenital transmission and determinants of seroprevalence and seroconversion in early childhood.

## Methods

### Study design

**Fig. 1:**
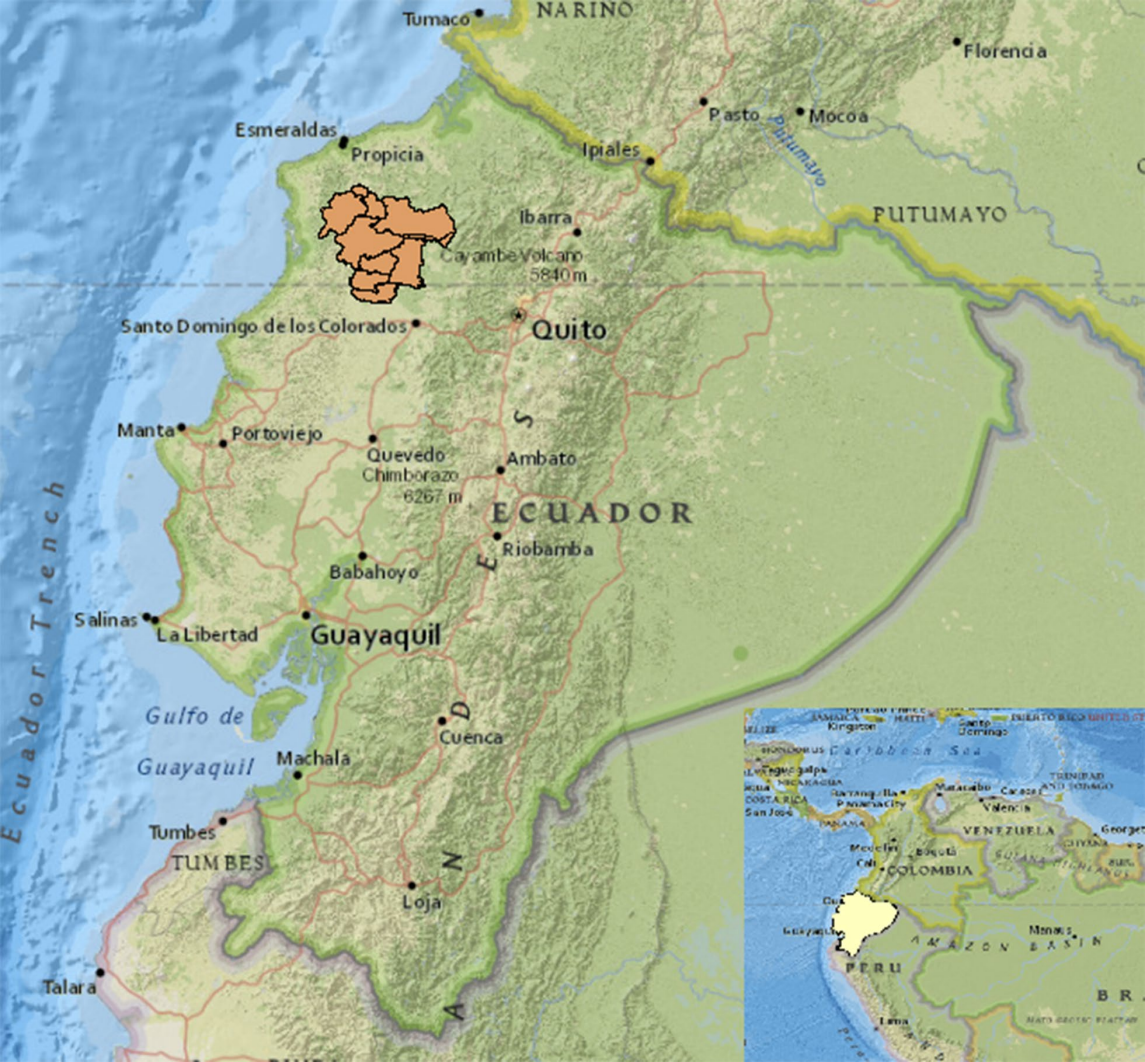
Map of the study area in the district of Quinindé, Esmeraldas Province, Ecuador

The present study was nested within a larger birth cohort, the ECUAVIDA cohort, of 2404 mother-child pairs followed from birth to 5 years of age in the district of Quinindé in Esmeraldas Province, Ecuador, that has been described in detail elsewhere [5]. Quinindé District, which is largely rural, is in a tropical region of coastal Ecuador (Fig. 1), and has a mean annual temperature of 30 ºC and relative humidity of 80%; the district has a population of 88,000 inhabitants, who live below 150 m altitude. Main sources of income are cultivation of palm oil and tropical fruits, and timber extraction. Mother-child pairs for the analysis were selected on the basis of availability of a maternal blood and cord blood sample. Data and samples were collected at around the time of birth of the child and at 7 and 13 months, and 2, 3 and 5 years.

### Data and sample collection

Risk factor data were collected by maternal questionnaire, blood samples were collected for plasma, and stool samples were collected for parasite microscopy using a combination of direct saline wet mounts, modified Kato-Katz, and formol-ether concentration methods [6]. Anthelmintic treatment was provided to mothers with soil-transmitted helminth (STH) infections after delivery and to children with positive stools for STH parasites, as described [6]. *Toxocara* spp. immunoglobulin G (IgG) antibodies were measured in plasma using a standardized in-house indirect enzyme-linked immunosorbent assay with *T. canis* larvae excretory-secretory antigens, and a pre-adsorption step against *Ascaris lumbricoides* antigens, as described [7]. A pool of sera from 10 *Toxocara* spp.-infected subjects was used as the positive control and a pool of 14 *Toxocara* spp.-uninfected subjects (i.e. children without history of contact with dogs and cats) was used as the negative control. The cut-off for positivity was the mean optical density of negative control wells + 3 SDs.

### Statistical analysis

For statistical analysis, *Toxocara* spp. seropositivity, defined as a binary longitudinal repeated-measures outcome, was investigated for potential associations with relevant covariates, some of which were time-varying (i.e. cat and dog exposures in the child’s household and STH infections). Population average models were fit to these binary outcomes using generalized estimating equations (GEE), as described [8, 9], with the assumption of missing at random. Seroconversion rates (%) of infection were determined for each time period between sampling as the proportion of seronegative children who became positive. Logistic regression models were used to explore associations between seroconversion rates at 2, 3 and 5 years and potential risk factors in preference to a single GEE model that gave unreliable estimates. For logistic regression models, cumulative exposures to domestic pets or STH up to the analysis time point were used as covariates. Multivariable models were constructed with backward elimination from an initial model containing all variables with *P* < 0.1 in univariate analyses. Statistical significance was inferred by *P* < 0.05. Analyses were done using Stata version 11.

## Results

**Fig. 2:**
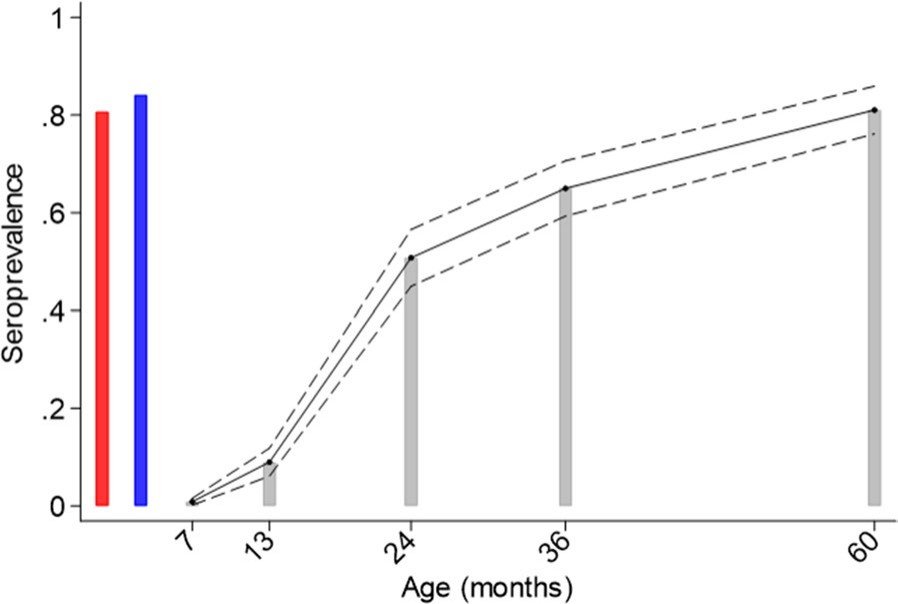
Anti-*Toxocara* spp. immunoglobulin G seroprevalence in mothers and in children from birth to 60 months of age. Shown are results from mothers during the third trimester of pregnancy (*red bar*), cord blood (*blue bar*) and children at 7, 24, 36, and 60 months (*grey bars*) of age. Actual (*bars*) and predicted (*continuous line* with 95% confidence intervals as* dashed lines*) seroprevalence are shown

**Fig. 3:**
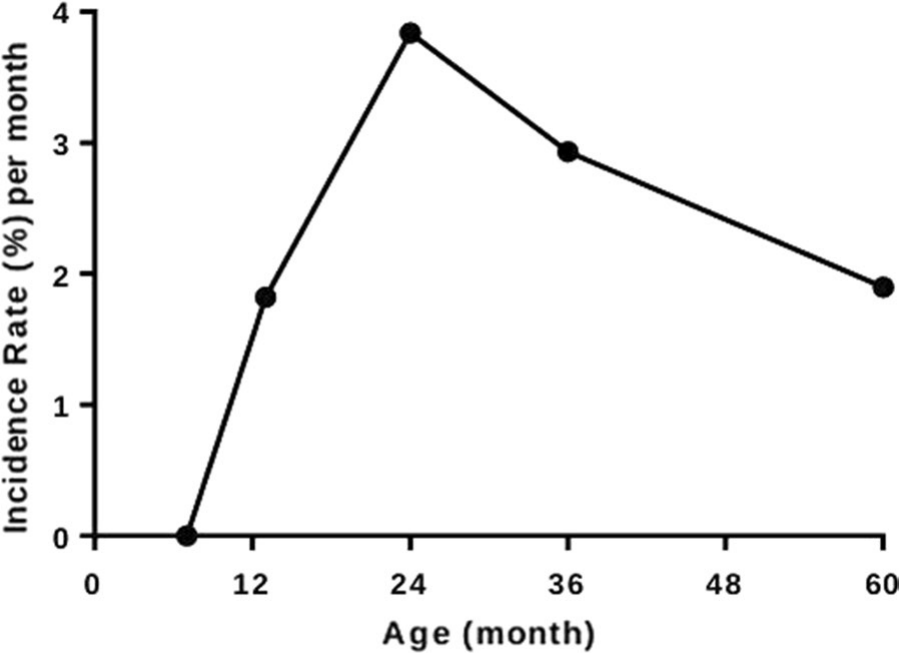
Monthly seroconversion rates at 7, 13, 24, 36 and 60 months of age

Plasma samples were analysed for 290 mothers and their children at birth (cord blood, 290/290), for 267 (92.1%) samples collected at 7 months, and for 267 (92.1%), 261 (90.0%), 268 (92.4%), and 247 (85.2%) samples collected at 13 months, and 2, 3 and 5 years, respectively. Seropositivity for *Toxocara* spp. IgG was 80.7% in mothers, 84.1% in cord blood, and 0% at 7 months, 10.9% at 13 months, 49.0% at 2 years, 65.7% at 3 years, and 81.0% at 5 years (Fig. 2). Distributions of potential risk factors and frequencies of *Toxocara* spp. seropositivity at 2, 3 and 5 years are shown in Table 1. Overall crude and adjusted associations with potential risk factors over the observation period are shown also in Table 1. Variables significantly associated with seroprevalence in adjusted analyses were: age [Odds ratio (OR) 2.06, 95% confidence interval (CI) 1.66–2.56, *P* < 0.001], male sex (female vs. males, OR 0.66, 95% CI 0.48–0.89, *P* = 0.006), maternal ethnicity (non-Afro-Ecuadorian vs. Afro-Ecuadorian, OR 0.65, 95% CI 0.47–0.91, *P* = 0.011), lower maternal educational level (completed primary vs. illiterate, OR 0.61, 95% CI 0.40–0.93, *P* = 0.021; completed secondary vs. illiterate, OR 0.57, 95% CI 0.34–0.96, *P* = 0.034), socioeconomic level (average vs. low, OR 0.62, 95% CI 0.43–0.89, *P* = 0.010; high vs. low, OR 0.60, 0.40–0.89, *P* = 0.009), and a childhood STH infection (OR 2.29, 95% CI 1.51–3.47, *P* < 0.001). Variables included in the multivariable model but not reaching statistical significance were maternal *Toxocara* spp. seropositivity (OR 1.46, 95% CI 0.97–2.13, *P* = 0.064) and household cats (OR 1.40, 95% CI 0.99–1.97, *P* = 0.055). Seroconversion rates were: 10.9% (29/267) between 7 and 13 months, 42.2% (95/225) between 13 months and 2 years, 35.1% (47/134) between 2 and 3 years, and 45.7% (37/81) between 3 and 5 years. Seroconversion rates per month/years were 1.8%, 3.8%, 2.9%, and 1.9% at 13 months, 2, 3 and 5 years of age, respectively (Fig. 3). Few children reverted to seronegative once seropositive: none of 24 children seropositive at 13 months became negative at 2 years (among children for whom results were available at both 13 months and 2 years), 3 of 128 (2.3%) positive children at 2 years became negative at 3 years, and 3 out of 176 (1.7%) positive children at 3 years were negative at 5 years. Significant determinants of seroconversion in adjusted analyses were: between 13 months and 2 years, having any STH infection [adjusted (adj.) OR 2.80, 95% CI 1.38–5.70, *P* = 0.004]; between 2 and 3 years, male sex (female vs. male, adj. OR 0.37, 95% CI 0.17–0.78, *P* = 0.009) and more frequent reports of domestic cats (> =  4 reports vs. 0–1 report, adj. OR 5.20, 95% CI 1.13–23.89, *P* = 0.034); and between 3 and 5 years, any STH infection (adj. OR 3.46, 95% CI 1.22–9.81, *P* = 0.019).

**Table 1:** Frequencies of risk factors and *Toxocara* spp. seropositivity in children at 2, 3 and 5 years of age showing age-adjusted and multivariable associations of risk factors with overall anti-*Toxocara* spp. immunoglobulin G seropositivity over the observation period

Variable	Category	2 Years (*n* = 261)		3 Years (*n* = 268)		5 Years (*n* = 247)		Age-adjusted		Multivariable effects	
		Overall	% Positive	Overall	% Positive	Overall	% Positive	OR (95%CI)	*p*-value	OR (95% CI)	*p*-value
Age	Age							1.77 (1.39–2.27)	< *0.001*	2.06 (1.66–2.56)	< *0.001*
Maternal seropositivity	No	51 (19.5%)	45.1%	53 (19.8%)	54.7%	46 (18.6%)	71.7%	1		1	
	Yes	210 (80.5%)	50.0%	215 (80.2%)	68.4%	201 (81.4%)	83.1%	1.69 (1.18–2.42)	0.004	1.46 (0.97–2.13)	*0.064*
Sex	Male	121 (46.4%)	50.4%	127 (47.4%)	72.4%	117 (47.4%)	86.3%	1		1	
	Female	140 (53.6%)	47.9%	141 (52.6%)	59.6%	130 (52.6%)	76.2%	0.65 (0.49–0.87)	0.004	0.66 (0.48–0.89)	*0.006*
Maternal age (years)	≤ 20	67 (25.7%)	50.8%	72 (26.9%)	72.2%	69 (27.9%)	85.5%	1			
	21–29	129 (49.4%)	49.6%	132 (49.3%)	65.9%	121 (49.0%)	79.3%	0.89 (0.63–1.26)	0.505		
	≥ 30	65 (24.9%)	46.2%	64 (23.9%)	57.8%	57 (23.1%)	79.0%	0.69 (0.46–1.04)	0.073		
Maternal ethnicity	Afro-Ecuadorian	81 (31.0%)	55.6%	84 (31.3%)	69.1%	78 (31.6%)	82.1%	1		1	
	Non-Afro Ecuadorian	180 (69.0%)	46.1%	194 (68.7%)	64.1%	169 (68.4%)	80.5%	0.73 (0.53–0.99)	*0.047*	0.65 (0.47–0.91)	*0.011*
Maternal educational level	Illiterate	43 (16.5%)	55.8%	49 (18.3%)	75.5%	44 (17.8%)	86.4%	1		1	
	Completed primary	167 (64.0%)	50.3%	167 (35.1%)	63.5%	156 (63.2%)	82.7%	0.61 (0.40–0.90)	*0.014*	0.61 (0.40–0.93)	*0.021*
	Completed secondary	51 (19.5%)	39.2%	52 (19.4%)	63.5%	47 (19.0%)	70.2%	0.44 (0.27–0.71)	*0.001*	0.57 (0.34–0.96)	*0.034*
Socioeconomic status^a^	Low	90 (34.5%)	56.7%	93 (34.7%)	69.9%	86 (34.8%)	88.4%	1		1	
	Medium	91 (35.3%)	50.0%	94 (35.1%)	62.8%	84 (34.0%)	78.6%	0.61 (0.43–0.86)	*0.005*	0.62 (0.43–0.89)	*0.010*
	High	79 (30.3%)	39.2%	81 (30.2%)	64.2%	77 (31.2%)	75.3%	0.50 (0.35–0.72)	< *0.001*	0.60 (0.40–0.89)	*0.009*
Household dogs	No	187 (71.7%)	48.7%	186 (70.2%)	62.9%	117(48.6%)	77.8%	1			
	Yes	74 (28.4%)	50.0%	79 (29.8%)	70.9%	124(51.5%)	83.9%	1.27 (0.92–1.75)	*0.140*		
Household cats	No	203 (77.8%)	47.3%	193 (72.8%)	61.7%	120(49.8%)	79.2%	1		1	
	Yes	58 (22.2%)	55.2%	72 (22.2%)	75.0%	121(50.2%)	82.6%	1.39 (1.00–1.93)	*0.051*	1.40 (0.99–1.97)	*0.055*
Household dogs and/or cats	No	157 (60.2%)	47.1%	143 (54.0%)	59.4%	78 (32.4%)	78.2%	1			
	Yes	104 (39.8%)	51.9%	122 (46.0%)	72.1%	163 (67.6%)	82.2%	1.32 (0.99–1.77)	*0.063*		
Maternal STH	No	139 (53.7%)	44.6%	136 (51.1%)	61.8%	126 (51.4%)	74.6%	1			
	Yes	120 (46.3%)	54.2%	130 (48.9%)	69.2%	119 (48.6%)	87.4%	1.58 (1.19–2.12)	*0.002*		
Childhood STH	No	234 (90.7%)	44.9%	212 (80.6%)	61.8%	165 (68.2%)	75.2%	1		1	
	Yes	24 (9.3%)	83.3%	51 (19.4%)	78.4%	77 (31.8%)	92.2%	2.55 (1.71, 3.83)	< *0.001*	2.29 (1.51–3.47)	< *0.001*
Area of residence	Urban	180 (68.9%)	48.3%	183 (68.3%)	66.1%	170 (68.8%)	82.4%	1			
	Rural	81 (31.1%)	50.6%	85 (31.7%)	64.7%	77 (31.2%)	77.9%	0.96 (0.70–1.30)	0.777		
Household overcrowding^b^	No	149 (57.1%)	45.6%	153 (57.1%)	63.4%	140 (56.7%)	45.6%	1			
	Yes	81 (42.9%)	53.6%	115 (42.9%)	68.7%	107 (43.3%)	53.6%	1.41 (1.05–1.89)	*0.021*		
Bathroom type	Latrine	170 (65.1%)	51.8%	176 (65.7%)	68.8%	159 (64.4%)	83.0%	1			
	Water Closet	91 (34.9%)	44.0%	92 (34.3%)	59.8%	88 (35.6%)	77.3%	0.72 (0.53–0.97)	*0.029*		

Not shown are covariates Age^2^ [unadjusted Odds ratio (OR) 0.987, 95% confidence interval (CI) 0.979–0.994, *P* = 0.001; adjusted OR 0.982, 95% CI 0.975–0.989, *P* < 0.001] and Age^3^ (unadjusted OR 1.0001, 95% CI 1.00004–1.0002, *P* < 0.001; adjusted OR 1.00015, 95% CI 1.0001–1.0002, *P* < 0.001), both of which were included in the multivariable model. ORs and 95% CIs were estimated using generalized estimating equations

*P* < 0.05* in italics*

^a^Socioeconomic status represents tertiles of* z*-scores obtained using a factor analysis

^b^Household overcrowding is defined as > 3 people per sleeping room

## Discussion

In the present analysis, we used a birth cohort recruited in a rural district in a tropical region of coastal Ecuador to address two questions relating to the epidemiology of human toxocariasis: whether infection can occur through congenital transmission, and what are the risk factors for the acquisition of infections during childhood. We followed a sample of 290 newborns longitudinally until 8 years of age and showed that at 7 months of age none of the children studied had evidence of anti-*Toxocara* spp*.* IgG antibodies, indicating that congenital transmission from seropositive and presumably infected mothers is unlikely to occur in this setting. The acquisition of anti-*Toxocara* spp. IgG antibodies was associated with childhood STH infections, male sex, maternal ethnicity, and household poverty.

We observed a high seroprevalence for toxocariasis in our study population of approximately 80% in mothers and in children by 5 years of age. The seroprevalence observed in this population is as at the upper level of that observed previously in other low and middle-income countries [10]. While seropositivity does not imply active infection—*Toxocara* spp. larvae have been estimated to survive in a human host for 2–4 years [11–13]—the fact that few children reverted to seronegative over the course of the study (< 2.5%/year) indicates that reinfections were common in this setting.

Vertical transmission of toxocariasis as a consequence of larval migration via the placenta has been suggested to occur following exposure to infection during pregnancy or through re-activation of dormant larvae in a chronically infected woman undergoing the immunological changes associated with pregnancy [2]. The only previous report of potential *in utero* transmission in humans was in a newborn of a seropositive mother, who developed a *Toxocara*-like lesion in the eye, which was detected by ultrasound and disappeared after chemotherapy [3]. Although highly suggestive, the latter report does not provide unequivocal evidence for congenital transmission: definitive diagnosis requires detection of larvae or larval DNA in tissue or body fluid samples. Transmission of *T. canis* in dogs, a natural host, is primarily through transplacental and transmammary transmission [4], with re-activation of dormant larvae occurring during pregnancy [14]. Congenital transmission has been documented also in paratenic hosts such as mice through sequestration of larvae in the brain [4]. In the present study two observations did not favour congenital transmission: although seropositivity in cord blood was equivalent to that in mothers (representing transplacental transfer of maternal IgG to foetus), anti-*Toxocara* spp. IgG antibodies became undetectable by 7 months of age in all children as the infants cleared maternal antibodies—after 7 months, seroconversion occurred rapidly and reached maternal levels of seropositivity by 5 years of age; maternal seropositivity was not strongly associated with seroprevalence or seroconversion in the cohort.

The few previous longitudinal studies on children that estimated seroconversion rates for anti-*Toxocara* spp. IgG antibodies observed cohorts of children for 1 year: (1) a study in Campinas in São Paulo State in Brazil, done in a population with 28% seroprevalence among children, followed 72 seronegative children aged 6–14 years and estimated an incidence of 7.6% (or 0.6%/month) [15]; (2) another study from Campinas, done in a population with 14.6% seroprevalence, followed 77 seronegative children aged 2–12 years and observed a seroconversion rate of 10.4% (or 0.9%/month) [13]; and (3) a study in the city of Assis Brazil, in Acre State in Brazil [16], done in a population with 24% seroprevalence in children, followed 228 children aged 6 months to 12 years and observed seroconversion rates of up to 13.9% (or 1.2%/month) depending on the age of the children [16]. These seroconversion rates are slightly lower than those observed in the present study (average of 1.3%/month), in which we observed children over a 5-year period from birth. However, in the present study, the risk of acquiring infection was greatest during the second (3.8% seroconversion rate/month) and third (2.9%/month) years of life.

*Toxocara* spp. infections during early childhood are likely to be acquired in and around the household. Sources of infection are domestic dogs and cats [11, 17–19] in the context of poor hygiene and open contamination of the peri-domestic environment with pet faeces [20]. Infected pet faeces can contaminate play areas of young children and drinking water of households without potable water [16]. Peri-domestic environments are often more heavily contaminated with *Toxocara* spp. eggs than recreational areas (e.g. parks and public play areas) [20, 21]. Male sex is a recognized risk factor in many settings [11, 17, 18], and is likely a reflection of gender-determined patterns of play, as are co-infections with STH through shared risk factors of poor environmental hygiene [7, 17, 22]. We have documented previously, in a different population in coastal Ecuador, the presence of *Toxocara* spp. DNA in floor and mattress dust samples taken inside households [23]—mattress samples were positive more frequently than floor samples, and the presence of *T. canis* DNA was more frequently detected than *T. cati* DNA (23% vs. 4% of samples, respectively) [23]. Contamination of beds could occur through pets grooming themselves in sleeping areas: infection rates among cats may be very high in some settings [24], and embryonated *Toxocara* spp. eggs can be detected in pet fur at much higher densities than found in soil [25–28]. The relative importance of dog versus cat transmission in a specific setting may vary by infection rates among pets and local customs with respect to entry of pets into houses. In the present study, exposure to cats was a non-significant risk factor for seroprevalence, while cumulative cat exposures, but not dog exposures, were strongly associated with seroconversion in children between 2 and 3 years of age.

This study had a number of important limitations. We inferred infection by the presence of anti-*Toxocara* spp*.* IgG antibodies using a well-validated serological assay. Such assays may be less specific in populations exposed to other helminth infections such as *A. lumbricoides* with which *Toxocara* spp. share significant immunologic cross-reactivity [1, 7]. Despite pre-adsorption of test sera against a phosphate buffered saline extract of *A. lumbricoides* adult worms to minimize serological cross-reactions [7], we cannot exclude false positive reactions relating to other STH co-infections. Use of assays such as Western blot to improve specificity is useful for individual diagnosis but less practical for epidemiological surveys. Although childhood STH infections were an important risk factor for *Toxocara* spp. seropositivity, this observation is perhaps most likely explained by shared risk factors and uncontrolled confounding. The sample size was relatively small, limiting our ability to identify risk factors with relatively small effects and infrequent outcomes such as allergy. The sample was sufficient, however, to show that congenital transmission, if it does occur, is an unusual occurrence in this population (i.e.  < 1 in 200). Important strengths were the longitudinal design from birth, frequent sampling and data collection allowing age-dependent seroconversion rates to be estimated, the collection of data on a wide range of potential risk factors and confounders, and high rates of follow up in excess of 85% at all sampling time points which would have minimized selection bias.

## Conclusions

In conclusion, to our knowledge, this is the first study to use a birth cohort to examine the acquisition of anti-*Toxocara* spp. IgG antibodies during the first 5 years of life in an area of high endemicity in a tropical setting. Our data suggest that if congenital transmission does occur in this setting it is a rare occurrence, and that important risk factors for acquisition of infection are household cats, male sex, and concurrent STH infections indicating poor hygiene.

## Data Availability

Data are available upon request.
